# Age of first self-harm act in childhood and adolescence: A scoping review protocol

**DOI:** 10.12688/hrbopenres.13764.1

**Published:** 2023-07-26

**Authors:** Daisy Wiggin, Elaine McMahon, Fiona McNicholas, Almas Khan, Eve Griffin

**Affiliations:** 1 School of Public Health, University College Cork, Cork, Ireland; 2 National Suicide Research Foundation, Cork, Ireland; 3 Department of Psychiatry, School of Medicine, University College Dublin, Dublin, Ireland; 4 Department of Child and Adolescent Liaison Psychiatry, Our Lady’s Children’s Hospital Crumlin, Dublin, Ireland; 5 Lucena Child and Adolescent Mental Health Services, St John of God, Dublin, Ireland

**Keywords:** Youth self-harm, onset of self-harm, child and adolescent mental health, suicidal behaviour

## Abstract

**Background**: Self-harm in young people is associated with adverse outcomes for many. The age of first self-harm is not often reported in the literature and there is considerable heterogeneity in how it is reported and in the methods used to estimate it. The objective of this study will be to examine the age of first self-harm act in childhood and adolescence and to identify the methods used to assess this.

**Methods**: This scoping review will follow JBI guidance. Five electronic databases, Medline, PsycInfo, CINAHL Plus, Embase, and Web of Science will be searched from inception. Grey literature will be searched via Google Scholar. Studies reporting the age of first act of self-harm in young people aged 17 years and younger are of interest. Any study design and methodology will be eligible for inclusion. Included studies may use any self-harm definition, any measures used to assess self-harm and the age of the first act. The focus can be in any context, including health services presenting or community samples. Title and abstract screening and full text screening will be carried out by two reviewers independently. The data extraction tool will be piloted by two reviewers independently, included studies will undergo data extraction by one reviewer and this will be checked by a second, independent reviewer.

**Results**: The resulting data will be presented using descriptive statistics, in tabular format, and accompanied with a narrative presentation of results. The results of this study will be distributed by publication in an academic journal.

## Introduction

Self-harm in childhood and adolescence is a concerning public health phenomenon associated with an increased risk of mental health problems, future self-harm ( Mars *et al.,* 2014) and dying by suicide ( Hawton *et al.,* 2020; Ross *et al.,* 2023). Self-harm is defined in this review as ‘an act with non-fatal outcome in which an individual deliberately initiates a non-habitual behaviour, that without intervention from others will cause self-harm, or deliberately ingests a substance in excess of the prescribed or generally recognised therapeutic dosage, and which is aimed at realising changes that the person desires via the actual or expected physical consequences’ ( Schmidtke *et al.,* 1996). This definition does not differentiate based on the motive(s) of or suicidal intent associated with the behaviour. It is argued that suicidal intent is a dimensional phenomenon ( Hawton *et al.,* 2012) and therefore, it is inclusive of both non-suicidal self-injury (NSSI) and attempted suicide, a dichotomy used mostly in the United States and Canada ( Muehlenkamp *et al.,* 2012). Moreover, an international study of self-harm in the community found that ‘non-suicidal’ reasons (e.g., relief from terrible state of mind, self-punishment) often co-occurred with a wish to die ( Scoliers *et al.,* 2009).

The lifetime prevalence of self-harm in adolescence has been estimated to be 16.1%, however there are inconsistencies in estimates which are thought to arise from differences in nomenclature, geographical area, and whether a single or multiple item measure is used ( Muehlenkamp *et al.,* 2012). In Ireland, rates of hospital-presenting self-harm have increased by 22% in adolescents and young people under 25 years of age, with the most pronounced increase among females aged 10–14 years ( Griffin *et al.,* 2018), suggesting that the age of engaging in self-harm for the first time may be decreasing.

Defining and measuring age of onset of psychopathology in general is difficult ( Jones, 2013), the knock-on effect being that the operationalisation of age of onset in the literature is highly inconsistent. In a large meta-analysis estimating the age of onset of a range of disorders, first symptom, first diagnosis, first hospitalisation and first contact with treatment service were reported definitions ( Solmi *et al.,* 2022). Similarly, in schizophrenia, age at onset has been defined as age at first admission to hospital, age at first psychotic symptoms, and age at first contact with healthcare professionals ( Immonen *et al.,* 2017). It is difficult to know if early symptoms are in fact signalling a mental disorder, it is only in retrospect when a decision can be made regarding the relevance of early symptoms to an eventual disorder ( Jones, 2013). Self-harm may be different, as it is a behaviour rather than a disorder, however it is often associated with borderline personality disorder for example ( American Psychiatric Association, 2022), although its close relationship with this diagnosis has been challenged ( Long *et al.,* 2013). Self-harm will be considered as a standalone behaviour in this review. A prior systematic review found that age at first NSSI act averaged between 12–14 years; variations in the estimate suggest that there may be different developmental trajectories in NSSI ( Cipriano *et al.,* 2017). Further, a meta-analysis estimates the first self-harm act to be at an average age of 12.8 years (95% CI 11.78-13.84; Gillies *et al.,* 2018). However, the operationalisation of age of first act and the methods used to determine first act are not reported in these reviews.

The study of age of first act of self-harm is relatively new, and an informal literature search suggests studies in the area are rather heterogeneous in their definitions, operationalisations, and methods of assessment. Therefore, a scoping review is an appropriate design to provide an overview of the literature with respect to the age of first act of self-harm in childhood and adolescence ( Munn *et al.,* 2018). This review aims to report as well as map the available evidence with a view to improving understanding of how age of first self-harm act is measured and defined, and to identify gaps in how this can be accurately achieved. This review aims to answer the following research question and sub question: What is the age of first act of self-harm in young people aged 17 years and younger and what definitions and methods are used to determine age of first act?

## Methods

This protocol was prepared following JBI guidance for the preparation of scoping review protocols ( Peters *et al.,* 2022) and has been registered on the Open Science Framework ( Wiggin *et al.,* 2023). The review will be conducted in accordance with JBI guidance for the conduct of scoping reviews ( Peters *et al.,* 2020) and reported in line with the Preferred Reporting Items for Systematic Reviews and Meta-Analyses Scoping Review extension (PRISMA-ScR; Tricco *et al.,* 2018).

### Eligibility criteria

All primary studies which report the age of first self-harm act will be eligible for inclusion. No language or time restrictions will be applied. Table 1 contains a list of the full inclusion and exclusion criteria. An exclusion criterion of note is stereotypic self-harm behaviours associated with intellectual disabilities and neurodivergence. These behaviours are argued to be biologically driven and serve a purpose of self-stimulation and a need for increased sensory input (e.g., intense, repetitive, rhythmic behaviours such as eyeball pressing and head banging; ( Ryan *et al.,* 2008). This category of behaviour is outside the conceptual scope of this review.

**Table 1.** Inclusion and exclusion criteria.

Inclusion Criteria	Exclusion Criteria
▪ Sample first self-harmed at 17 years or younger ▪ Age of first self-harm act reported ▪ Any self-harm definition used by authors ▪ Any measures used to assess age of first self-harm act ▪ Any context (e.g., health-service presenting, community)	▪ Age of first self-harm act not reported ▪ Age of first self-harm act was reported to be in adulthood (18 years and above) ▪ Study examines stereotypic self-harm behaviours more common in intellectual disabilities and autism (e.g., intense, repetitive, rhythmic behaviours such as eyeball pressing and head banging) ▪ Study reports on thoughts of self-harm and/or thoughts of suicide only

### Population

The population of interest is anyone who first self-harmed during childhood and adolescence. Adolescence as the time between childhood and adulthood has varied timespans and beginning and ending ages. With evolving economic and social contexts, it has recently been argued that the ages of 10–24 years better fit the current development of adolescents ( Sawyer *et al.,* 2018). That being said, health services are provided to children and adolescents until the age of 18 years in many countries, after which they are treated as adults. Therefore, the term adolescence in this review refers to those aged 10–17, with children being aged 10 years and under ( Sawyer *et al.,* 2018).

### Concepts

The concepts of interest are self-harm, as defined above, and the age at which self-harm was first engaged in during childhood and adolescence. Self-harm is defined differently across the literature, with some drawing a dichotomy between suicide attempts and NSSI and others who do not use this dichotomy. Additionally, NSSI Disorder and Suicidal Behavior Disorder have been proposed in the DSM-5 ( American Psychiatric Association, 2022). Studies operating from any definition of self-harm or these proposed disorders will be eligible for inclusion in this review. Age of first act is also differentially applied in the literature; as previously mentioned it can be defined as the first self-harm presentation to health services or when first self-harmed in a private setting. Studies will be eligible regardless of the operationalisation of age of first act; details of the operationalisation will be collected as part of this review. However, this can be impacted by the availability of data and methodological constraints which are discussed next.

### Context

Self-harm can occur in the context of community and in health service-presenting samples, including primary and secondary care. The iceberg model of self-harm estimates that adolescents who present to hospital represent 6% of those who self-harm in the community in Ireland ( McMahon *et al.,* 2014); this estimate is higher in England ( Geulayov *et al.,* 2018). This provides evidence that hospital presentations represent a small proportion of adolescents who self-harm. In the interest of capturing a broad view of the literature, all settings are of interest in this current review. In a community sample, the start of self-harm can mean when the person initiates the behaviour in a private setting, presents to a general practitioner or other healthcare practitioner, or it is confirmed by a parent/guardian. This information is often captured using retrospective recall via self-report survey data or interviews. In self-harm that presents to hospital, the start of self-harm would often be captured by surveillance registries (e.g., National Self-Harm Registry Ireland ( Joyce *et al.,* 2022), Multicentre Study of Self-Harm in England ( Hawton *et al.,* 2007)) as the first presentation to hospital. All determinations of engaging in self-harm for the first time will be included in this review and reported.

### Evidence sources

Electronic searches for relevant studies have been conducted in Medline (EBSCO), PsycInfo (EBSCO), Embase (Elsevier), CINAHL Plus (EBSCO), and Web of Science (Clarivate) from inception to 26 ^th^ June 2023. Grey literature will be included by screening the first 50 results of a Google Scholar search. Any study methodology will be eligible for inclusion. In the instance where sources are encountered in duplicate – primary sources and evidence syntheses that have included the primary source – primary sources will be excluded if already incorporated into an included evidence synthesis unless the data they contain are not otherwise reported in the evidence synthesis.

### Search strategy

Search terms related to the concepts of age at first act (e.g., ‘age of onset’), self-harm (e.g., ‘deliberate self-harm’) and the population of interest (e.g., ‘child’) were developed in consultation with prior systematic reviews and primary studies in this area. The full search strategy for Medline is available from the Open Science Framework registration ( Wiggin *et al.,* 2023). The search strategy was developed with and validated by a librarian in University College Cork.

### Study records

***Data management*.** The search results will be exported to Zotero for deduplication and then to Rayyan ( https://www.rayyan.ai/) for managing citations, performing title and abstract screening, and data extraction.

***Selection process*.** Titles and abstracts of eligible studies will be assessed according to the eligibility criteria in Table 1. The screening process will be piloted with five potentially relevant articles to assess for consistent application. Articles deemed relevant at this stage will undergo full-text screening according to the eligibility criteria. During the pilot and both screening stages, two reviewers will work independently, disagreements will be resolved by discussion or a third reviewer if necessary. Given the broad nature of a scoping review, new relevant terms and locations of evidence may be discovered during the selection process. Therefore, the search strategy may be modified during the review process to account for new discoveries. Studies excluded after full-text screening will be reported, alongside reasons for exclusion, in the final report. Included studies will be subject to forward and backward citation searching.

***Data extraction*.** Data extraction will be performed on all studies included after full-text screening. This will be completed by one reviewer and checked by an independent second reviewer. Disagreements will be resolved by discussion or a third reviewer if necessary. The data extraction form below will be piloted by two reviewers independently on a small subset of studies to ensure all relevant results are extracted, with any proposed discrepancies and amendments being discussed and decided by the wider research team. Study authors will be contacted for additional information if necessary. The final version of the data extraction form used will be included in the final report with any amendments explained. The proposed data extraction items are outlined in Table 2.

**Table 2.** Data extraction items.

Items
1. Primary author
2. Year of publication
3. Geographic setting(s) of the study
4. Funding source(s)
5. Study design
6. Study period
7. Self-harm definition used
8. First self-harm act definition used
9. Method used to determine age of first act
10. Method used to assess self-harm behaviour
11. First act is a primary study aim
12. Sample size
13. Sample characteristics:
• Age (mean and standard deviation or equivalent)
• Gender (proportions)
• Age of first act (mean and standard deviation or equivalent)
• Method of self-harm used for first act (proportions)
• Contact with primary and/or secondary mental health services (proportions)

### Data analysis and presentation

Findings will be descriptively, narratively, and graphically presented. Evidence related to age of first self-harm act will be reported using descriptive statistics and tables. Definitions of first self-harm act, methods used to assess age at first act, definitions of self-harm, and the relevant contexts will be presented in tabular format, narratively, and using descriptive statistics if appropriate.

## Deviations from the protocol

Amendments to the protocol prior to and during the conduct of the review will be documented by tabulating version history and important changes in the protocol. Any such deviations will be described in the final report.

## Study status

The search strategy has been implemented and records are awaiting screening at the time of publication of this protocol.

## Data Availability

Open Science Framework: Age of first self-harm act in childhood and adolescence: A scoping review protocol. https://doi.org/10.17605/OSF.IO/FEHTD. ( Wiggin *et al.,* 2023). This project contains the following underlying data: Medline-Search-Strategy_Age-of-Onset_ScR.docx. (Full search strategy for Medline). Data are available under the terms of the Creative Commons Attribution 4.0 International license (CC-BY 4.0).
