# Age of first self-harm act in childhood and adolescence: A scoping review protocol

**DOI:** 10.12688/hrbopenres.13764.2

**Published:** 2023-11-22

**Authors:** Daisy Wiggin, Elaine McMahon, Fiona McNicholas, Eve Griffin

**Affiliations:** 1 School of Public Health, University College Cork, Cork, Ireland; 2 National Suicide Research Foundation, Cork, Ireland; 3 Department of Psychiatry, School of Medicine, University College Dublin, Dublin, Ireland; 4 Department of Child and Adolescent Liaison Psychiatry, Our Lady’s Children’s Hospital Crumlin, Dublin, Ireland; 5 Lucena Child and Adolescent Mental Health Services, St John of God, Dublin, Ireland

**Keywords:** Youth self-harm, onset of self-harm, child and adolescent mental health, suicidal behaviour

## Abstract

**Background:**

Self-harm in youth is associated with adverse outcomes for many. The age of first self-harm is not often reported in the literature and there is considerable heterogeneity in how it is reported and in the methods used to estimate it. The objective of this study will be to examine the age of first self-harm act in childhood and adolescence and to identify the research methods used to assess this.

**Methods:**

This scoping review will follow JBI guidance. Five electronic databases, Medline, PsycInfo, CINAHL Plus, Embase, and Web of Science will be searched from inception. Grey literature will be searched via Google Scholar. Studies reporting the age of first act of self-harm in young people aged 17 years and younger are of interest. Any study design and methodology will be eligible for inclusion. Included studies may use any self-harm definition, any measures used to assess self-harm and the age of the first act. The focus can be in any context, including health services presenting or community samples. Title and abstract screening and full text screening will be carried out by two reviewers independently. The data extraction tool will be piloted by two reviewers independently, included studies will undergo data extraction by one reviewer and this will be checked by a second, independent reviewer.

**Results:**

The resulting data will be presented using descriptive statistics, in tabular format, and accompanied with a narrative presentation of results. The results of this study will be distributed by publication in an academic journal.

## Introduction

Self-harm in childhood and adolescence is a concerning public health phenomenon associated with an increased risk of psychological distress, future self-harm ( Mars *et al.,* 2014) and dying by suicide ( Hawton *et al.,* 2020; Ross *et al.,* 2023). Self-harm is defined in this review as ‘an act with non-fatal outcome in which an individual deliberately initiates a non-habitual behaviour, that without intervention from others will cause self-harm, or deliberately ingests a substance in excess of the prescribed or generally recognised therapeutic dosage, and which is aimed at realising changes that the person desires via the actual or expected physical consequences’ ( Schmidtke *et al.,* 1996). This definition does not differentiate based on the motive(s) of or suicidal intent associated with the behaviour. It is argued that suicidal intent is a dimensional phenomenon ( Hawton *et al.,* 2012) and therefore, it is inclusive of both non-suicidal self-injury (NSSI) and attempted suicide, a dichotomy used mostly in the United States and Canada ( Muehlenkamp *et al.,* 2012). Moreover, an international study of self-harm in the community found that ‘non-suicidal’ reasons (e.g., relief from terrible state of mind, self-punishment) often co-occurred with a wish to die ( Scoliers *et al.,* 2009).

The lifetime prevalence of self-harm in adolescence has been estimated to be 16.1%, however there are inconsistencies in estimates which are thought to arise from differences in nomenclature, geographical area, and whether a single or multiple item measure is used ( Muehlenkamp *et al.,* 2012). In Ireland, rates of hospital-presenting self-harm have increased by 22% in adolescents and young people under 25 years of age, with the most pronounced increase among those aged 10–14 years at 75% ( Griffin *et al.,* 2018), suggesting that the age of engaging in self-harm for the first time may be decreasing.

Age of onset is widely studied in the context of medical diagnoses. Determining the age of onset of mental disorders (referred to as emotional or psychological distress henceforth; Division of Clinical Psychology, 2015) can be challenging ( Jones, 2013), the knock-on effect being that the operationalisation of age of onset in the literature is highly inconsistent. In a large meta-analysis estimating the age of onset of emotional distress, first complaint, first diagnosis, first hospitalisation and first contact with intervention service were reported definitions ( Solmi *et al.,* 2022). Self-harm is often associated with significant emotional and/or psychological distress, with 31.3% and 23.6% of young people aged 10–19 years also experiencing low mood and anxiety respectively ( Morgan *et al.*, 2017; Supplementary appendix 1). These proportions, while substantial, leave a majority of young people who self-harm who not been given psychiatric diagnoses. On this basis, self-harm will be considered as a standalone behaviour in this review. A prior systematic review found that age at first NSSI act averaged between 12–14 years; variations in the estimate suggest that there may be different developmental trajectories in NSSI ( Cipriano *et al.,* 2017). Further, a meta-analysis estimates the first self-harm act to be at an average age of 12.8 years (95% CI 11.78-13.84; Gillies *et al.,* 2018). However, the operationalisation of age of first act and the methods used to determine first act are not reported in these reviews.

The study of age of first act of self-harm is relatively new, and an informal literature search suggests studies in the area are rather heterogeneous in their definitions, operationalisations, and research methods of assessment. Therefore, a scoping review is an appropriate design to provide an overview of the literature with respect to the age of first act of self-harm in childhood and adolescence ( Munn *et al.,* 2018). This review aims to report as well as map the available evidence with a view to improving understanding of how age of first self-harm act is measured and defined, and to identify gaps in how this can be accurately achieved. This review aims to answer the following research question and sub question: What is the age of first act of self-harm in young people aged 17 years and younger and what definitions and research methods are used to determine age of first act?

## Methods

This protocol was prepared following JBI guidance for the preparation of scoping review protocols ( Peters *et al.,* 2022) and has been registered on the Open Science Framework ( Wiggin *et al.,* 2023). The review will be conducted in accordance with JBI guidance for the conduct of scoping reviews ( Peters *et al.,* 2020) and reported in line with the Preferred Reporting Items for Systematic Reviews and Meta-Analyses Scoping Review extension (PRISMA-ScR; Tricco *et al.,* 2018).

### Eligibility criteria

All primary studies which report the age of first self-harm act will be eligible for inclusion. No language or time restrictions will be applied. Table 1 contains a list of the full inclusion and exclusion criteria. An exclusion criterion of note is stereotypic self-harm behaviours associated with intellectual disabilities and neurodivergence. These behaviours are argued to be biologically driven and serve a purpose of self-stimulation and a need for increased sensory input (e.g., intense, repetitive, rhythmic behaviours such as eyeball pressing and head banging; ( Ryan *et al.,* 2008). This category of behaviour is outside the conceptual scope of this review.

**Table 1.** Inclusion and exclusion criteria.

Inclusion Criteria	Exclusion Criteria
▪ Sample first self-harmed at 17 years or younger ▪ Age of first self-harm act reported ▪ Any self-harm definition used by authors ▪ Any measures used to assess age of first self-harm act ▪ Any context (e.g., health-service presenting, community, school, third sector)	▪ Age of first self-harm act not reported ▪ Age of first self-harm act was reported to be in adulthood (18 years and above) ▪ Study examines stereotypic self-harm behaviours more common in intellectual disabilities and autism (e.g., intense, repetitive, rhythmic behaviours such as eyeball pressing and head banging) ▪ Study reports on thoughts of self-harm and/or thoughts of suicide only ▪ Study protocols, editorials, letters to the editor, case reports, case series

### Population

The population of interest is anyone who first self-harmed during the period of childhood and adolescence. Adolescence as the time between childhood and adulthood has varied timespans and beginning and ending ages. With evolving economic and social contexts, it has recently been argued that the ages of 10–24 years better fit the current development of adolescents ( Sawyer *et al.,* 2018). Currently mental health services are provided to young people until the age of 18 years in many European countries participating in the MILESTONE study with some exceptions being younger than this, after which they are treated as adults; these countries were Austria, Belgium, Bulgaria, Croatia, Czech Republic, Denmark, Estonia, Finland, Germany, Greece, Hungary, Ireland, Italy, Latvia, Lithuania, Luxembourg, the Netherlands, Poland, Portugal, Romania, Slovakia, Slovenia, Spain, Sweden, United Kingdom ( Signorini *et al.,* 2017). Therefore, the term adolescence in this review refers to those aged 10–17, with children being aged 10 years and under ( Sawyer *et al.,* 2018).

### Concepts

The concepts of interest are self-harm, as defined above, and the age at which self-harm was first engaged in during childhood and adolescence. Self-harm is defined differently across the literature, with some drawing a dichotomy between suicide attempts and NSSI and others who do not use this dichotomy. Additionally, ‘NSSI Disorder’ and ‘Suicidal Behavior Disorder’ have been proposed in the DSM-5 ( American Psychiatric Association, 2022). Studies operating from any definition of self-harm or these proposed diagnostic categories will be eligible for inclusion in this review. Age of first act is also differentially applied in the literature; as previously mentioned it can be defined as the first self-harm presentation to health services or when first self-harmed in a private setting. Studies will be eligible regardless of the operationalisation of age of first act; details of the operationalisation will be collected as part of this review. However, this can be impacted by the availability of data and methodological constraints which are discussed next.

### Context

Self-harm can occur in community and healthcare contexts, which might lead to help-seeking at primary and secondary healthcare settings, school settings and third sector organisations. The iceberg model of self-harm estimates that adolescents who present to hospital represent 6% of those who self-harm in the community in Ireland ( McMahon *et al.,* 2014); this estimate is higher in England ( Geulayov *et al.,* 2018), providing evidence that hospital presentations represent a small proportion of adolescents who self-harm. In the interest of capturing a broad view of the literature, any setting where self-harm occurs or young people who self-harm may be supported are of interest in this current review. In a community sample, the start of self-harm can mean when the person initiates the behaviour in a private setting, presents to a general practitioner or other healthcare practitioner, or it is confirmed by a parent/guardian. This information is often captured using retrospective recall via self-report survey data or interviews. In self-harm that presents to hospital, the start of self-harm would often be captured by surveillance registries (e.g., National Self-Harm Registry Ireland ( Joyce *et al.,* 2022), Multicentre Study of Self-Harm in England ( Hawton *et al.,* 2007)) as the first presentation to hospital. All determinations of engaging in self-harm for the first time will be eligible for inclusion in this review and reported.

### Evidence sources

Electronic searches for relevant studies have been conducted in Medline (EBSCO), PsycInfo (EBSCO), Embase (Elsevier), CINAHL Plus (EBSCO), and Web of Science (Clarivate) from inception to 26 ^th^ June 2023. Grey literature will be included by screening the first 100 results of a Google Scholar search, searching EThOS, BASE, and the World Health Organisation’s publications related to suicide and suicide prevention. Additionally, the following government, charity, and third sector organisation’s publications sections of their websites will be searched: the Health Service Executive’s Connecting for Life, the National Suicide Research Foundation, Jigsaw, Samaritans, Mental Health Foundation, and the Agency for Healthcare Research and Quality. Any study methodology will be eligible for inclusion. In the instance where sources are encountered in duplicate – primary sources and evidence syntheses that have included the primary source – primary sources will be excluded if already incorporated into an included evidence synthesis unless the data they contain are not otherwise reported in the evidence synthesis. The findings reported in any synthesised evidence will be checked against the originating primary study.

### Search strategy

Search terms related to the concepts of age at first act (e.g., ‘age of onset’), self-harm (e.g., ‘deliberate self-harm’) and the population of interest (e.g., ‘child’) were developed in consultation with prior systematic reviews and primary studies in this area. The full search strategy for Medline is available from the Open Science Framework registration ( Wiggin *et al.,* 2023). The search strategy was developed with and validated by a librarian in University College Cork.

### Study records

***Data management*.** The search results will be exported to Zotero for deduplication and then to Rayyan ( https://www.rayyan.ai/) for managing citations and performing title and abstract and full-text screening. Data extraction will be managed in Microsoft Excel. Given the minimal exclusion criteria for this review, there is a chance that the screening and extraction processes will become lengthy and burdensome. In this event, a third reviewer will be invited to join the team.

***Selection process*.** Titles and abstracts of eligible studies will be assessed according to the eligibility criteria in Table 1. The screening process will be piloted with five potentially relevant articles to assess for consistent application. Articles deemed relevant at this stage will undergo full-text screening according to the eligibility criteria. During the pilot and both screening stages, two reviewers will work independently, disagreements will be resolved by discussion or a third reviewer if necessary. Given the broad nature of a scoping review, new relevant terms and locations of evidence may be discovered during the selection process. Therefore, the search strategy may be modified during the review process to account for new discoveries. Studies excluded after full-text screening will be reported, alongside reasons for exclusion, in the final report. Included studies will be subject to forward and backward citation searching.

***Data extraction*.** Data extraction will be performed on all studies included after full-text screening. This will be completed by one reviewer using Microsoft Excel, the extracted data will then be checked against the originating record by an independent second reviewer. Disagreements will be resolved by discussion or a third reviewer if necessary. The data extraction form below ( Table 2) will be piloted by two reviewers independently on a small subset of studies to ensure all relevant results are extracted, with any proposed discrepancies and amendments being discussed and decided by the wider research team. Study authors will be contacted for additional information if necessary. The final version of the data extraction form used will be included in the final report with any amendments explained.

**Table 2.** Data extraction items.

Items
1. Primary author
2. Year of publication
3. Geographic setting(s) of the study
4. Funding source(s)
5. Study design
6. Study period
7. Self-harm definition used
8. First self-harm act definition used
9. Method used to determine age of first act
10. Method used to assess self-harm behaviour
11. First act is a primary study aim
12. Sample size
13. Sample characteristics:
• Age (mean and standard deviation or equivalent)
• Gender (proportions)
• Age of first act (mean and standard deviation or equivalent)
• Method of self-harm used for first act (proportions)
• Contact with primary and/or secondary mental health services (proportions)

### Data analysis and presentation

Findings will be descriptively, narratively, and graphically presented. Evidence related to age of first self-harm act will be reported using descriptive statistics and tables if appropriate. Definitions of first self-harm act, methods used to assess age at first act, definitions of self-harm, and the relevant contexts will be presented in tabular format, narratively, and using descriptive statistics if appropriate.

## Implications for research and practice

It is anticipated that the results of this review will inform future research examining age of first act of self-harm by highlighting gaps, contextual nuance, and inconsistencies in the current state of the literature. Regarding clinical relevance, as the review will be sensitive to context i.e. hospital, primary care, school, and third sector, we intend for the findings to offer exploratory insight into the age at which young people start self-harm for those working across a variety of contexts.

## Deviations from the protocol

Amendments to the protocol prior to and during the conduct of the review will be documented by tabulating version history and important changes to the protocol. Any such deviations will be described in the final report.

## Study status

The search strategy has been implemented and records are awaiting screening at the time of publication of this protocol.

## Data Availability

Open Science Framework: Age of first self-harm act in childhood and adolescence: A scoping review protocol. https://doi.org/10.17605/OSF.IO/FEHTD. ( Wiggin *et al.,* 2023). This project contains the following underlying data: Medline-Search-Strategy_Age-of-Onset_ScR.docx. (Full search strategy for Medline). Data are available under the terms of the Creative Commons Attribution 4.0 International license (CC-BY 4.0).
