# Supplementary material for: Age of first self-harm act in childhood and adolescence: A scoping review protocol
Source: HRB Open Res. 2023 Nov 22;6:36. Originally published 2023 Jul 26. [Version 2] doi: 10.12688/hrbopenres.13764.2 (PMC10714104; doi:10.12688/hrbopenres.13764.2)
Supplement: Peer review history

# Reviewer report: Reviewer response for version 2

**DOI:** 10.21956/hrbopenres.15130.r37143

**Published:** 2023-12-11

**Authors:** Faraz Mughal

**Affiliations:** 1 School of Medicine, Keele University, Keele, England, UK

I am happy with these revisions. Please proceed to full approval from my part.

Is the study design appropriate for the research question?

Yes

Is the rationale for, and objectives of, the study clearly described?

Yes

Are sufficient details of the methods provided to allow replication by others?

Yes

Are the datasets clearly presented in a useable and accessible format?

Yes

Reviewer Expertise:

NA

I confirm that I have read this submission and believe that I have an appropriate level of expertise to confirm that it is of an acceptable scientific standard.

# Reviewer report: Reviewer response for version 2

**DOI:** 10.21956/hrbopenres.15130.r37142

**Published:** 2023-11-23

**Authors:** Daniel Romeu

**Affiliations:** 1 Leeds Institute of Health Sciences, University of Leeds, Leeds, England, UK

I have reviewed the revised article and I can confirm that the authors have considered my comments and made appropriate improvements to their manuscript. I am happy for the article to be accepted for indexing in its current form.

Is the study design appropriate for the research question?

Yes

Is the rationale for, and objectives of, the study clearly described?

Yes

Are sufficient details of the methods provided to allow replication by others?

Yes

Are the datasets clearly presented in a useable and accessible format?

Not applicable

Reviewer Expertise:

NA

I confirm that I have read this submission and believe that I have an appropriate level of expertise to confirm that it is of an acceptable scientific standard.

# Reviewer report: Reviewer response for version 1

**DOI:** 10.21956/hrbopenres.15056.r36098

**Published:** 2023-10-05

**Authors:** Faraz Mughal

**Affiliations:** 1 School of Medicine, Keele University, Keele, England, UK

This is a clear, concise, and well written protocol. 

 I only have a few comments to make as it is written very well, is coherent, and should add useful new findings to this topic area.

 It is good the authors will report this scoping review in line the relevant PRISMA checklist. 

 I would expand the google scholar search to the first 100 or even more hits to screen and consider using incognito to get an accurate picture of what it is on the internet in terms of grey literature. Could also run searches on Microsoft Edge.

 It is not clear how the data extraction will be checked by a second member of the team - what is this process?

 A minor point but I couldn't find the MEDLINE search strategy in the OSF registration.

Is the study design appropriate for the research question?

Yes

Is the rationale for, and objectives of, the study clearly described?

Yes

Are sufficient details of the methods provided to allow replication by others?

Yes

Are the datasets clearly presented in a useable and accessible format?

Yes

Reviewer Expertise:

Primary care, suicide prevention, self-harm.

I confirm that I have read this submission and believe that I have an appropriate level of expertise to confirm that it is of an acceptable scientific standard, however I have significant reservations, as outlined above.

# Response

**Published:** 2023-11-17

**Authors:** Daisy Wiggin

**Affiliations:** University College Cork, Ireland

Thank you for reviewing our protocol and offering valid points for improvement. We respond to your comments below.

**Comment**: “I would expand the google scholar search to the first 100 or even more hits to screen and consider using incognito to get an accurate picture of what it is on the internet in terms of grey literature.”

***Response*** *: This is a valid point. We will expand the search of Google Scholar to include the first 100 results. In addition, as per the suggestion of Reviewer 1, we’ve included searching EThOS to improve our coverage of grey literature , including publications from charity organisations, NGOs, governmental agencies, and dissertations. The two abovementioned changes have been reflected and detailed in the ‘Evidence sources’ subsection of the Methods section.*

**Comment**: “It is not clear how the data extraction will be checked by a second member of the team - what is this process?”

***Response*** *: The text in the ‘Data extraction’ part of the ‘Study records’ subsection of the Methods section has been updated to include that extracted data will be checked against the originating record.*

**Comment**: “A minor point but I couldn't find the MEDLINE search strategy in the OSF registration.”

***Response*** *: We apologise that you weren’t able to access the Medline search strategy, we hope you can access it directly via the following link:* *https://osf.io/uqmkf* *.*

# Reviewer report: Reviewer response for version 1

**DOI:** 10.21956/hrbopenres.15056.r36097

**Published:** 2023-10-05

**Authors:** Daniel Romeu

**Affiliations:** 1 Leeds Institute of Health Sciences, University of Leeds, Leeds, England, UK

Thank you for the opportunity to review this scoping review protocol. It is clearly and comprehensively written and addresses an important gap in the literature. I support its acceptance for indexing with a few minor suggested amendments.

**Introduction**

 The background to the proposed review is clearly outlined and its rationale is evident. Although this is only the protocol, it would be useful for the authors to outline the expected implications of this review and its results. What impact will this knowledge have on future research and clinical practice?

 The authors mention the association of self-harm with borderline personality disorder and allude to its controversy without offering opinion. The concept of borderline personality disorder is in itself controversial. Self-harm is associated with a breadth of mental health conditions (mood disorders, psychotic disorders, neurodevelopmental conditions, substance misuse, personality disorders etc). I wonder whether the authors should comment on this (rather than the association with BPD specifically) and use that as justification for the consideration of self-harm as a standalone behaviour in this review.

**Methods**

 The authors have transparently described the proposed methods which are robust and in keeping with JBI guidance. The process of study selection and data extraction is strong and its iterative nature has been acknowledged. The authors plan to pilot the tools they will use for these processes.

Eligibility criteria

 The justification of excluding young people aged 18 and over is unclear beyond explanation that this is the cut-off for adult services in "many countries". Specifying which countries have this cut-off will improve the generalisability and impact of this review and its findings.

 The authors refer to settings as either being community- or health service-based, then explain that "all settings" are of interest. I suspect that this refers to "all healthcare settings" but this is not explicit. There is no mention of other important settings such as schools and third-sector organisations which should be considered. 

Evidence sources

 The empirical evidence sources look appropriate and the search strategies appear broad but robust. The protocol would benefit from commentary on the feasibility of managing the results given the minimal exclusion criteria (have pilot searches been conducted)?

 The authors have specified Google Scholar as their only grey literature source, and they will stop screening after 50 results. Reviewing the grey literature is essential to the achieving the research aims and the breadth of this topic area warrants a more extensive approach. Have the authors considered a 'stop rule' for their Google search (stopping when X number of irrelevant consecutive results have been found)? Charities, third-sector organisations and dissertations may yield important results, and this review would be strengthened by the authors' consideration of these sources of information. Have they considered grey literature databases e.g. SCIE, EThOS?

Is the study design appropriate for the research question?

Yes

Is the rationale for, and objectives of, the study clearly described?

Yes

Are sufficient details of the methods provided to allow replication by others?

Yes

Are the datasets clearly presented in a useable and accessible format?

Not applicable

Reviewer Expertise:

Mental health, self-harm, health services, unexplained symptoms

I confirm that I have read this submission and believe that I have an appropriate level of expertise to confirm that it is of an acceptable scientific standard, however I have significant reservations, as outlined above.

# Response

**Published:** 2023-11-17

**Authors:** Daisy Wiggin

**Affiliations:** University College Cork, Ireland

Thank you for your thoughtful review of our protocol and proposing important opportunities for clarification and improvement. We have provided responses to your comments below.

**Comment:** “it would be useful for the authors to outline the expected implications of this review and its results. What impact will this knowledge have on future research and clinical practice?”

***Response:*** *Thank you for this suggestion, we have added a section after the methods entitled ‘Implications for research and practice’ in which we describe the anticipated impact of this research.*

**Comment:** “The authors mention the association of self-harm with borderline personality disorder and allude to its controversy without offering opinion. The concept of borderline personality disorder is in itself controversial. Self-harm is associated with a breadth of mental health conditions (mood disorders, psychotic disorders, neurodevelopmental conditions, substance misuse, personality disorders etc). I wonder whether the authors should comment on this (rather than the association with BPD specifically) and use that as justification for the consideration of self-harm as a standalone behaviour in this review.”

***Response*** *: Thank you for this helpful comment. This is a very valid point, and we agree that self-harm is associated with a wider breadth of emotional distress than originally mentioned. We have revised the Introduction section accordingly. In paragraph three of the Introduction section, we mention that a substantial proportion of young people who self-harm have been given psychiatric diagnoses, but a majority self-harm in the absence of receiving a diagnosis. We outline that this is the basis on which we decide to treat self-harm as a standalone behaviour.*

**Comment:** “The justification of excluding young people aged 18 and over is unclear beyond explanation that this is the cut-off for adult services in “many countries”. Specifying which countries have this cut-off will improve the generalisability and impact of this review and its findings.”

***Response:*** *In the ‘Population’ subsection of the Methods section, we list countries who use 18 years of age as the transition age from child and adolescent to adult mental health services according to the MILESTONE study.*

**Comment: **“The authors refer to settings as either being community- or health service-based, then explain that “all settings” are of interest. I suspect that this refers to “all healthcare settings” but this is not explicit. There is no mention of other important settings such as schools and third-sector organisations which should be considered.”

***Response:*** *Thank you for highlighting this. We intend to include all settings including healthcare and community settings like schools and third sector organisations and have clarified this in the ‘Context’ subsection and in ‘Table 1. Inclusion and exclusion criteria’ located in the Methods section.*

**Comment:** “The protocol would benefit from commentary on the feasibility of managing the results given the minimal exclusion criteria (have pilot searches been conducted)?”

***Response:*** *The search strategy as detailed in this protocol retrieved approximately 3,000 original records on 26 ^th^ June 2023. We deem this a feasible number of records to screen over the course of a few months. If the screening and extraction process becomes lengthy we will invite a third reviewer to join the reviewing team. We have included text to this effect in the ‘Data management’ part of the ‘Study records’ subsection in the Methods section.*

**Comment:** “Reviewing the grey literature is essential to the achieving the research aims and the breadth of this topic area warrants a more extensive approach. Have the authors considered a ‘stop rule’ for their Google search (stopping when X number of irrelevant consecutive results have been found)? Charities, third-sector organisations and dissertations may yield important results, and this review would be strengthened by the authors’ consideration of these sources of information. Have they considered grey literature databases e.g. SCIE, EThOS?”

***Response:*** *Thank you for highlighting the importance of grey literature. We did not use a ‘stop rule’ for the Google Scholar search. SCIE is part of the Web of Science’s Core Collection, a database we included in our evidence sources. We will include EThOS in our grey literature search, expand our Google Scholar search to the first 100 results as per the suggestion of Reviewer 2, and have detailed how we will further expand our search to include the suggested sources of information in the ‘Evidence sources’ subsection of the Methods section.*
